# Growth-rate analysis of clathrin assembly: inferring endocytic kinetics in living cells and tissues

**DOI:** 10.3389/fmolb.2026.1844575

**Published:** 2026-06-24

**Authors:** Aritra Mondal, Comert Kural

**Affiliations:** 1 Interdisciplinary Biophysics Graduate Program, The Ohio State University, Columbus, OH, United States; 2 Department of Physics, The Ohio State University, Columbus, OH, United States

**Keywords:** clathrin, deep learning, endocytosis, fluorescence microscopy, growth rates

## Abstract

Measuring protein kinetics in living cells remains challenging due to the complexity of intracellular environments and the limitations of live-cell imaging. Clathrin-mediated endocytosis (CME) provides a model system in which protein assembly dynamics encode functional outcomes, yet conventional approaches rely on reconstructing full trajectories of individual events, making them sensitive to tracking errors and difficult to extend to dense or *in vivo* systems. Here, we review growth rate–based analysis of clathrin assembly as an alternative framework for quantifying endocytic kinetics. By extracting local rates of fluorescence intensity change from short temporal segments, this approach bypasses trajectory reconstruction and instead yields growth rate distributions that robustly capture ensemble dynamics. The standard deviation of these distributions serves as a quantitative metric of kinetic variability and distinguishes distinct dynamical regimes. We highlight how growth-rate analysis reveals key regulatory mechanisms, including membrane tension–dependent modulation of CME and spatiotemporal heterogeneity across cells and tissues, and discuss recent advances leveraging deep learning to infer dynamic behavior from single images. Together, these developments establish a scalable and minimally invasive strategy for measuring protein kinetics in living systems under physiologically relevant conditions.

## Introduction

Clathrin-mediated endocytosis (CME) is a central pathway by which cells internalize membrane components, extracellular ligands, and signaling receptors, thereby regulating processes such as nutrient uptake, membrane turnover, and signal transduction (McMahon and Emmanuel, 2011; Kaksonen and Roux, 2018; Kirchhausen et al., 2014). Clathrin-coated carriers account for a substantial portion of membrane trafficking between the plasma membrane and endosomal compartments, underscoring the fundamental role of this pathway in cellular organization. Consistent with this, disruption of core components of the clathrin machinery leads to severe developmental defects and embryonic lethality, highlighting its essential function *in vivo* (Chen et al., 2009; Ferguson et al., 2009; Mitsunari et al., 2005). Moreover, dysregulation of CME has been implicated in a wide range of human diseases, including cancer, muscle disorders, and neurological conditions such as Alzheimer’s disease (Bitoun et al., 2005; Dalgliesh et al., 2010; Harold et al., 2009; Kan et al., 2010; Mishra et al., 2002; Treusch et al., 2011; Wettey et al., 2002).

The process begins with the recruitment of adaptor proteins that nucleate the assembly of clathrin into a lattice at the plasma membrane, forming clathrin-coated pits (CCPs) (Brodsky et al., 2001; Kirchhausen, 2000). These structures progressively deform the membrane and, with contributions from accessory proteins such as actin and dynamin, undergo scission to generate intracellular vesicles (Kural et al., 2012; Cocucci et al., 2014; Boulant et al., 2011). As such, CME represents a tightly coordinated interplay between molecular assembly, membrane mechanics, and cytoskeletal forces.

A defining feature of CME is its pronounced spatiotemporal heterogeneity. Membrane tension, lipid composition, and protein interactions vary across different regions of a cell, leading to substantial variability in both the structure and kinetics of clathrin-coated structures (Boulant et al., 2011; Ferguson et al., 2017; Djakbarova et al., 2021; Willy et al., 2021a). As a result, CME dynamics cannot be fully captured by single-event descriptions but instead require quantitative frameworks that account for this variability.

Over the past decades, quantitative analysis of CME has relied heavily on tracking individual clathrin-coated structures over their full lifecycles (Mettlen and Danuser, 2014). These approaches aim to extract kinetic parameters such as initiation rates, lifetimes, and maturation dynamics, which are tightly linked to functional outcomes. However, accurate measurement of these parameters in living cells remains challenging due to the crowded intracellular environment, limited spatial and temporal resolution, phototoxicity, and errors in particle detection and tracking.

These limitations become particularly severe in dense cellular regions and *in vivo* systems, where reliable trajectory reconstruction is often infeasible. This has motivated the development of alternative approaches that do not rely on complete tracking of individual events. Instead, these methods quantify CME dynamics through local fluorescence intensity fluctuations and ensemble statistics, providing a more robust and scalable framework for measuring protein kinetics in living cells both *in vitro* and *in vivo*. Importantly, these approaches are complementary to—not replacements for—trajectory-based methods: they provide ensemble-level information in regimes where frame-to-frame particle linking fails, while single-particle tracking remains the method of choice for event-resolved questions such as productive-versus-abortive classification, mobility analysis, and dwell-time measurements at scission.

### Conventional approaches to quantifying CME dynamics and their limitations

**FIGURE 1:**
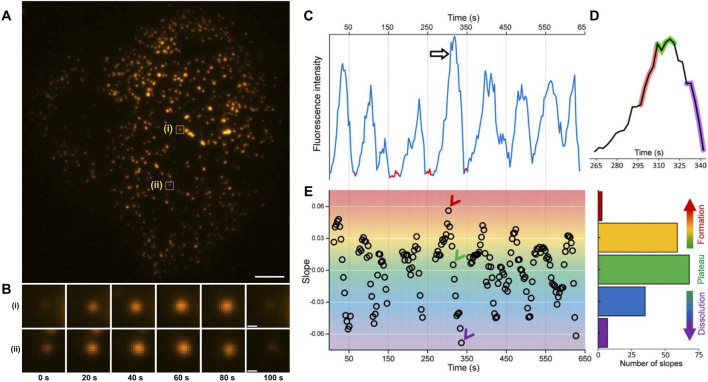
**(A)** Representative images of clathrin-coated vesicles on the ventral surface of SUM159 cells, imaged using TIRFM. Boxes (i) and (ii) indicate isolated clathrin coated vesicles (Scale bar-5µm) **(B)** Isolated clathrin coated vesicles tracked over time (Scale bar-300 nm). **(C)** Fluorescence intensity trace of a clathrin hotspot imaged at the ventral surface of a BSC-1 cell expressing AP2-GFP. Traces segments that are not used in growth rate calculation are shown in red, as they were considered the background signal. **(D)** Zoomed version of the clathrin-coated pit trace marked by the arrow in **(C)**. Shaded regions show 12-s-long fragments dwelling at formation (red), plateau (green), and dissolution (purple) phases of the pit (E, left) For the intensity trace in **(C)**, slope values representing the growth rates are determined from 12-s-long fragments centered on each time point. Red, green, and purple arrowheads mark the slopes corresponding to the growth, plateau, and dissolution fragments highlighted in **(D)**, respectively **(E)**, right Bar plot shows the distribution of the growth rates shown in the left panel. Positive and negative values correspond to formation and dissolution phases, respectively. Modified from Ferguson et al. (2016) [Creative Commons Attribution–NonCommercial–ShareAlike 3.0 Unported License (CC BY-NC-SA 3.0).] [Adapted from the original figure 1; an additional subfigure containing original data was added].

Quantitative analysis of CME has traditionally relied on live-cell fluorescence imaging—acquired predominantly using total internal reflection fluorescence (TIRF) microscopy for ventral plasma-membrane events and confocal or spinning-disk microscopy for apical and *in vivo* imaging (Figures 1A,B) — combined with single-particle tracking (Jaqaman et al., 2008; Mettlen and Danuser, 2014; Aguet et al., 2013; Kural and Kirchhausen, 2012; Kural et al., 2012). In these approaches, clathrin coat components such as clathrin light chain (CLC) or adaptor proteins (e.g., AP2) are fluorescently labeled and appear as diffraction-limited puncta. Puncta are detected by automated or semi-automated particle-detection algorithms [e.g., cmeAnalysis (Aguet et al., 2013), TrackMate (Tinevez et al., 2017; Ershov et al., 2022), or custom ImageJ/Fiji pipelines (Schindelin et al., 2012)], and the fluorescence intensity at each detected position is quantified either by 2D Gaussian fitting to the diffraction-limited spot—yielding integrated intensity—or by summing pixel values within a fixed radius after subtraction of a local background estimated from an annular region surrounding the spot (Thompson et al., 2002; Aguet et al., 2013). The fluorescence intensity of these puncta evolves over time, reflecting distinct stages of coat assembly, maturation, and disassembly (Figures 1C,D) (Ehrlich et al., 2004; Saffarian et al., 2009; Kural et al., 2012; Kural and Kirchhausen, 2012). By linking detections across consecutive frames, trajectories are reconstructed to extract kinetic parameters including lifetimes, peak intensities, and rates of assembly and disassembly.

**FIGURE 2:**
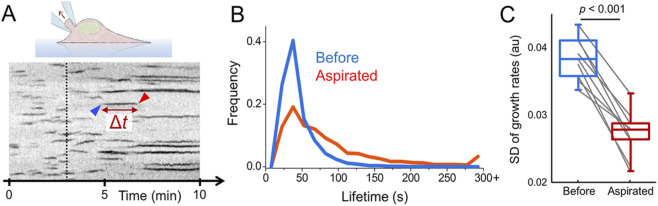
**(A)** Kymograph showing the clathrin activity at the ventral surface of a BSC1 cell expressing AP2–eGFP. Clathrin coat traces elongate gradually upon microaspiration (dashed line). Blue and red arrowheads mark the initiation and conclusion of an individual clathrin-coated structure, respectively. Δt indicates its lifetime **(B)** Clathrin coat lifetime distributions are shown for nine BSC1 cells imaged before and during microaspiration (ntraces = 40,943) **(C)** For the same 9 cells, the standard deviation of the clathrin growth rate distributions are shown in boxplots. Lines connect the standard deviation values obtained from the same cell before and during aspiration. The narrower growth rate distributions indicate slower clathrin coat dynamics. Modified from Ferguson et al. (2017) [Published by The Company of Biologists Ltd.; permission obtained from The Company of Biologists] [Adapted from selected parts of the original figure 1].

Among these metrics, the lifetime of clathrin-coated structures has been widely used as a proxy for endocytic activity. Shorter lifetimes are generally associated with more dynamic membrane regions and higher endocytic turnover, whereas longer lifetimes often indicate slower or inhibited dynamics. For example, increasing plasma membrane tension—such as through micropipette aspiration—raises the energetic barrier for membrane deformation, leading to reduced initiation rates and prolonged lifetimes of clathrin structures (Djakbarova et al., 2021; Akatay et al., 2022; Kural et al., 2024). These changes are readily reflected in shifts in lifetime distributions (Figures 2A,B).

The principal strength of single-particle tracking is that it preserves event-level information. By following individual clathrin-coated structures over their entire lifetimes, this approach can resolve heterogeneity between events, distinguish productive from abortive pits, estimate event-specific lifetimes, quantify mobility and diffusion, and link fluorescence intensity changes to specific stages of coat maturation. These features make single-particle tracking indispensable when the biological question depends on individual events. Recent advances have further extended the reach of single-particle tracking, including deep-learning-based detectors integrated into Fiji pipelines (Ershov et al., 2022), disassembly-asymmetry classifiers for distinguishing productive from abortive clathrin-coated pits (Wang et al., 2020), and Bayesian nonparametric frameworks such as BNP-Track that jointly infer emitter number, localization, and linking with quantified uncertainty—maintaining accuracy in dense particle fields where modular detect-then-link pipelines fail (Sgouralis et al., 2024).

Despite their utility, tracking-based approaches are intrinsically dependent on accurate detection and frame-to-frame association of individual puncta throughout their entire lifetimes. In practice, this requirement is difficult to satisfy. Errors arising from missed detections, false positives, merging and splitting of puncta, or premature termination of trajectories can introduce substantial biases in reconstructed lifetimes (Loerke et al., 2009; Aguet et al., 2013). These issues become particularly pronounced in densely populated regions, under low signal-to-noise conditions, or in highly dynamic cellular environments where particle identities are difficult to maintain.

Indeed, accurate quantification of CME dynamics using single-particle tracking has been estimated to require detection and tracking accuracies exceeding 99.9% (Mettlen and Danuser, 2014), a threshold that is rarely achievable in experimental datasets. The problem is especially acute during early stages of coat assembly, where fluorescence signals are weak and segmentation algorithms are prone to thresholding errors. More broadly, tracking performance deteriorates rapidly with decreasing signal-to-noise ratio, with significant losses in accuracy observed below SNR ≈4 (Chenouard et al., 2014; Smal et al., 2010).

In addition to computational limitations, the requirement to capture complete lifetimes imposes constraints on imaging. Continuous acquisition over extended periods increases photobleaching and phototoxicity, potentially perturbing the very dynamics being measured (Laissue et al., 2017). Attempts to mitigate these effects by reducing acquisition rates lead to temporal undersampling, which further compromises the ability to resolve rapid assembly and disassembly events. Together, these factors limit the applicability of traditional tracking-based approaches, particularly in complex or physiologically relevant settings such as tissues and *in vivo* systems.

### Growth rate–based analysis of CME dynamics

To address the limitations of trajectory-based analyses, alternative approaches have been developed that focus on short-timescale fluctuations in fluorescence intensity of clathrin coats (Ferguson et al., 2016). Growth-rate analyses use the same puncta detection and intensity-extraction steps described above (Gaussian fitting or fixed-radius averaging with local-background subtraction) but differ fundamentally in how the resulting temporal information is used. These methods leverage the characteristic temporal evolution of fluorescence signals associated with the recruitment and loss of clathrin-associated proteins. Rather than reconstructing full lifecycles, they extract local temporal derivatives of intensity traces, which directly report on instantaneous rates of coat assembly and disassembly.

In this framework, the slope of the fluorescence intensity trace at a given time point defines the growth rate of a clathrin coat (Figure 1E). Positive slopes correspond to assembly phases marked by protein recruitment, whereas negative slopes reflect disassembly. Near-zero slopes indicate intermediate or plateau phases, where net changes in coat composition are minimal. These local growth rates therefore provide a compact representation of the dynamic state of a clathrin coat without requiring knowledge of its full trajectory. In practice, slopes are computed from short trace fragments—typically 12 s long, centered on each time point (Figures 1D,E) — rather than over the entire ∼30–120 s lifetime of a clathrin-coated structure. This is the operational difference between growth-rate analysis and single-particle tracking: growth-rate analysis requires only that intensity be reliably measured over short, locally-linkable windows, not that a single coat be tracked from initiation to scission.

**FIGURE 3:**
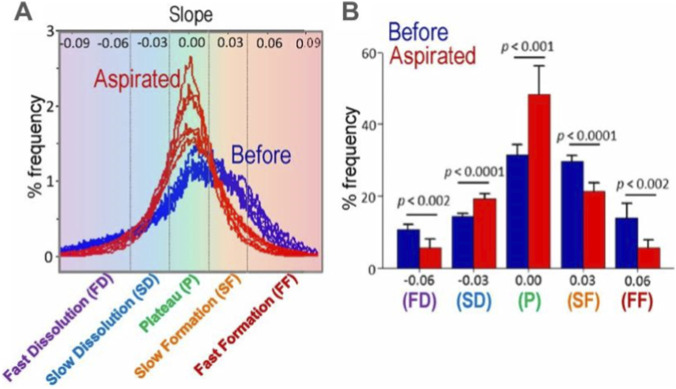
**(A)** Growth rate distributions are shown for seven different cells before and during aspiration. Change in CME dynamics induced by micropipette aspiration can be observed in growth rate distributions. In the control experiments (cells before aspiration), clathrin coats spend more time in the formation phase (i.e., the distribution is inclined toward positive slopes). The asymmetry was abolished upon aspiration and plateau phases got relatively longer (Ncells = 7 and Ntraces = 38136). **(B)** Growth rate distributions in B are assembled in five bins to better delineate different phases of clathrin-coated vesicle formation (FD, fast dissolution; FF, fast formation; P, plateau; SD, slow dissolution; SF, slow formation). The bars show mean +standard deviation to illustrate dispersion between cells. P-values were obtained using the twotailed t-test. Modified from Ferguson et al. (2016) [Creative Commons Attribution–NonCommercial–ShareAlike 3.0 Unported License (CC BY-NC-SA 3.0).] [Adapted from selected parts of the original figure 2].

By aggregating growth rates across many structures and time points, one can construct distributions that capture the ensemble dynamics of CME over a given interval. This shifts the analysis from single-event trajectories to population-level statistics, enabling robust characterization of endocytic activity. Because short fragmented traces—routinely discarded in trajectory-based analyses—can be retained, growth-rate datasets typically incorporate 10^4^–10^5^ trace fragments per condition (e.g., n_traces_ = 38,136 for 7 cells in Figure 3, 40,943 for 9 cells in Figure 2), an order of magnitude more than the number of complete trajectories normally retained after SPT quality filtering. Experimentally, these distributions are sensitive to physiological perturbations. For example, in BSC-1 cells, which predominantly form canonical clathrin-coated pits, growth rate distributions are enriched in positive values, reflecting active assembly dynamics. Increasing plasma membrane tension through micropipette aspiration suppresses these dynamics, leading to a redistribution toward smaller-magnitude growth rates and an increased prevalence of near-zero values (Boulant et al., 2011; Ferguson et al., 2016) (Figures 3A,B). This shift is consistent with slower coat turnover and prolonged lifetimes under high-tension conditions, demonstrating that growth-rate statistics can distinguish distinct kinetic regimes.

A key advantage of this approach is that it does not require continuous tracking of individual structures. Short or fragmented intensity traces, which are often discarded in conventional analyses, can be directly used to compute growth rates. This substantially increases the number of analyzable events and reduces biases associated with tracking errors. Moreover, growth-rate analysis is largely automated, with manual intervention typically limited to quality control, parameter selection during particle detection, and exclusion of poor imaging regions. As a result, growth-rate analysis remains robust in dense, noisy, or low signal-to-noise imaging conditions where trajectory reconstruction is unreliable.

Overall, growth-rate analysis is best understood as an ensemble-level framework that complements rather than replaces single-particle tracking, trading event-specific resolution for robustness and applicability on incomplete or fragmented traces.

### Spatial mapping of CME dynamics using clathrin growth rates

As described above, growth-rate distributions encode the local dynamic state of CME over short time intervals. Large-magnitude growth rates, both positive and negative, correspond to rapid assembly and disassembly events and therefore reflect highly dynamic endocytic activity. Consequently, the standard deviation (SD) of the growth-rate distribution provides a compact scalar measure of this variability. Systems exhibiting active CME display broader distributions and higher SD values, whereas conditions that suppress dynamics—such as elevated membrane tension in microaspirated BSC-1 cells—lead to narrower distributions and reduced SD [0.038 ± 0.003 (before aspiration) *versus* 0.027 ± 0.004 (during aspiration), P < 0.001] (Figure 2C) (Ferguson et al., 2016). Thus, the SD of growth rates serves as an interpretable metric for comparing kinetic regimes across different physiological conditions.

Importantly, this metric can be extended spatially to resolve heterogeneity in CME dynamics across the plasma membrane. By computing growth-rate statistics within localized regions—typically circular neighborhoods of 8-µm radius, chosen to balance spatial precision against the number of trace fragments needed for statistical convergence—SD values can be mapped as a function of position to generate spatial activity maps. These maps provide a direct link between local physical environments and protein kinetics, enabling visualization of how endocytic activity varies across a cell (Willy et al., 2017).

Application of this approach has revealed pronounced spatial heterogeneity in CME dynamics that correlates with underlying mechanical gradients. In particular, regions of elevated membrane tension exhibit reduced variability in growth rates, reflected by lower SD values, whereas regions under lower tension display enhanced dynamics (Boulant et al., 2011; Ferguson et al., 2017; Akatay et al., 2022; Kural et al., 2024). These observations establish SD-based analysis as a sensitive reporter of mechanoregulation in CME.

**FIGURE 4:**
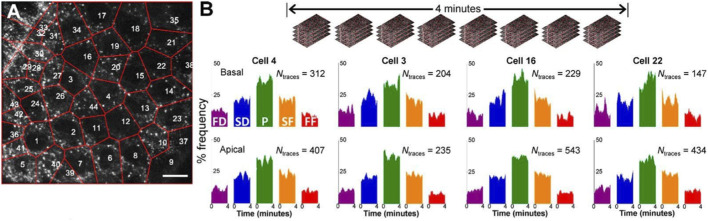
**(A)** Amnioserosa tissue of a late *Drosophila* embryo is imaged for 4 min using confocal z-stacks acquired every 3 s. Representative frame is an image section at the middle of a stack. Red lines represent the boundaries between amnioserosa cell centers, which are marked by numbers. Cell boundaries determined in each frame of a 3D time-lapse acquisition. Bar 10 µm. **(B)** Histograms show evolution of the growth rates corresponding to different cells selected from the amnioserosa tissue in **(A)**. Frequencies of the five phases are plotted as a function of time for the apical and basal surfaces. Modified from Ferguson et al. (2016) [Creative Commons Attribution–NonCommercial–ShareAlike 3.0 Unported License (CC BY-NC-SA 3.0).] [Adapted from selected parts of the original figure 9].

Notably, this framework extends beyond single-cell systems to more complex *in vivo* environments. In developing *Drosophila* embryos, growth-rate analysis applied to the amnioserosa (AS) reveals spatial variations in CME dynamics from short 4-min confocal z-stack recordings acquired every 3 s, despite challenges such as background noise and tissue motion (Ferguson et al., 2016) (Figures 4A,B). The ability to extract meaningful kinetic information from short imaging sequences is critical in these contexts, where long-duration tracking is impractical. These measurements suggest that endocytic activity is coordinated at the tissue level and influenced by local mechanical conditions.

**FIGURE 5:**
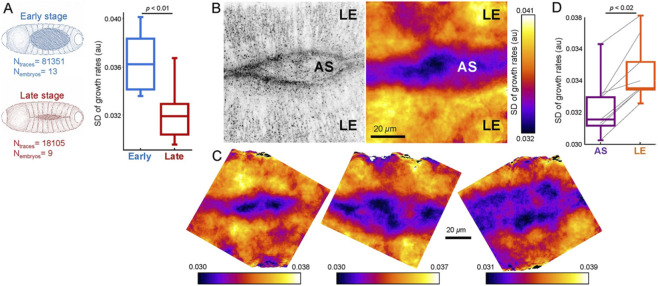
Spatiotemporal variations in clathrin dynamics can be detected within tissues of Drosophila embryo. **(A)** Clathrin dynamics slow down with increasing tension during late stages of the dorsal closure. Box plots show the SD of clathrin growth rate distributions obtained from early and late stage AS tissues. Reduced SD is a hallmark of slowed-down endocytosis. **(B)** Left, clathrin-coated structures at the dorsal surface of a *Drosophila* embryo. AS appears as a narrow opening between the two flanks of lateral epidermis (LE). Right, SD map of the clathrin growth rates obtained from the same area. The map is created by calculating the SD of apical clathrin growth rates within an 8-µm radius. Lower SD values in the AS region display slower clathrin dynamics with respect to the neighboring LE. **(C)** More examples demonstrating the heterogeneous clathrin dynamics at the dorsal surface of late *Drosophila* embryos. **(D)** Box plots show the SD of clathrin growth rate distributions obtained from LE and AS of eight embryos. Connected lines indicate the values obtained from the same embryo. Boxes extend to the quartiles, with a line at the median. Whiskers extend from the 10th to 90th percentiles. P values were obtained using the two tailed t-test. Modified from Willy et al. (2017) [Creative Commons Attribution–NonCommercial–ShareAlike 3.0 Unported License (CC BY-NC-SA 3.0).] [Reproduced from original figure 5].

Further insights arise during dorsal closure, a developmental process characterized by dynamic changes in tissue mechanics. As membrane tension increases in the AS, growth-rate analysis reveals a corresponding reduction in CME activity, reflected by lower SD values [0.036 ± 0.003 (early) *versus* 0.032 ± 0.002 (late), P < 0.01] (Figure 5A). Spatial mapping further demonstrates that CME dynamics are suppressed in the AS relative to the neighboring lateral epidermis (LE) (Figures 5B–D) (Willy et al., 2017). These findings highlight how mechanically distinct regions within the same tissue exhibit different endocytic behaviors and underscore the utility of SD-based metrics for probing protein kinetics across multiple spatial scales.

### Deep learning–based inference of CME dynamics

A recent downstream application of growth rate–based analysis leverages deep learning to infer CME dynamics directly from static fluorescence images. Both trajectory-based and growth-rate approaches require time-lapse imaging, albeit at different temporal resolutions, and are therefore subject to photobleaching, temporal undersampling, and residual tracking or segmentation errors. To overcome these limitations, a learning-based framework has been developed that predicts spatial maps of CME dynamics from single images, reducing the need for time-resolved acquisitions at the inference stage. Importantly, this framework is best understood as an *approximation* of growth-rate-derived SD maps—not a replacement for or independent measurement of CME dynamics—because it does not compute growth rates from time-lapse traces, but rather learns to reproduce the spatial patterns generated by growth-rate analysis.

In this approach, a convolutional neural network based on a modified U-Net architecture is trained on paired datasets consisting of static fluorescence images and corresponding SD maps derived from growth-rate analysis (Ronneberger et al., 2015; Wu and Kural, 2025). The dynamic information captured by the network is therefore inherited from growth-rate analysis during training, rather than measured directly from the static input. Through this training, the model learns to associate spatial patterns in clathrin fluorescence—reflecting local organization, density, and morphology—with underlying dynamic variability. In effect, the network maps static structural features to a proxy of kinetic activity.

**FIGURE 6:**
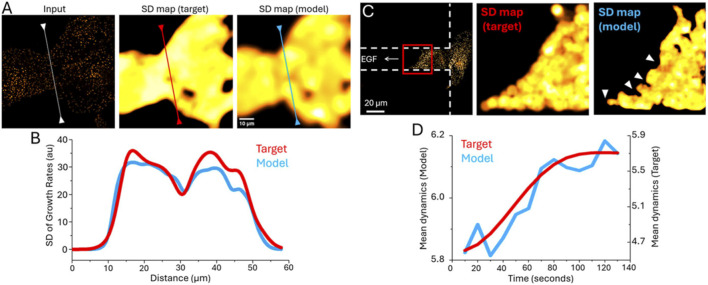
Deep learning accurately predicts spatial maps of CME dynamics from single images. **(A)** Representative input image (left), ground truth SD map derived from time-lapse tracking (middle), and predicted SD map from the trained U-Net model (right). The line profiles indicate the regions sampled for analysis in **(B)**. **(B)** Line intensity profiles of the SD of clathrin growth rates along the paths shown in **(A)**, comparing ground truth (red) and model prediction (blue). **(C)** Example of CME regulation in a cell stimulated with EGF. The red box highlights a region of interest enlarged to show ground truth SD map (left) and predicted SD map (right). The arrowheads denote areas at the cell edge where prediction errors occur, driven by a lack of clathrin-coated structures. **(D)** Mean CME dynamics (SD values) over time within the boxed region, showing concordance between ground truth (red) and model prediction (blue). The model captures spatial and temporal features of CME regulation from static input frames. Modified from Wu and Kural (2025) [Published under exclusive license by AIP Publishing; permission obtained from AIP Publishing as required.] [Reproduced from original figure 3].

Benchmarking demonstrates that the inferred SD maps closely recapitulate those obtained from growth-rate analysis, both in spatial organization and magnitude (Figure 6A). Quantitative comparisons, including line-profile analyses, confirm that the model accurately captures spatial variations in CME dynamics (Figure 6B). Notably, the framework generalizes to highly dynamic systems such as migrating cells, where it successfully reconstructs front–rear asymmetries in endocytic activity (Figures 6C,D). These results indicate that static fluorescence patterns encode sufficient information to infer underlying kinetic states.

This approach offers several practical advantages. By removing the requirement for time-lapse imaging at the inference stage, it minimizes photobleaching and phototoxicity, making it well suited for sensitive samples, including primary cells and *in vivo* systems. In addition, once trained, the model enables rapid and high-throughput analysis without manual intervention, in contrast to conventional tracking pipelines. However, performance depends on the quality and diversity of the training data, and accuracy can degrade in low-signal regions or under imaging conditions that deviate from the training distribution. Critically, because the training targets are themselves growth-rate-derived SD maps, the model inherits the assumptions and limitations of growth-rate analysis—including dependence on intensity calibration, photobleaching effects, sampling density, and the use of SD as a proxy for CME dynamics.

Overall, deep learning–based inference represents a natural extension of growth-rate analysis, transforming CME quantification from a temporally resolved measurement into a spatially encoded one. While it removes the need for time-lapse imaging at the inference stage, it remains fundamentally dependent on time-resolved growth-rate measurements for training and validation. More broadly, this approach points to a conceptual shift in which dynamic cellular processes can be inferred from static observations, opening new avenues for studying protein kinetics in complex biological systems.

### Limitations of growth rate–based analysis of CME dynamics

Despite its advantages over trajectory-based approaches, growth rate–based analysis has several limitations that should be considered. Most notably, its reliance on ensemble statistics means that it does not resolve the temporal progression of individual clathrin coats. While it robustly captures population-level dynamics, information about event-specific lifecycles—such as initiation-to-scission trajectories—is not directly accessible. Single-particle tracking therefore remains the primary methodology for questions that depend on event-specific information, including diffusion or mobility measurements and the classification of individual productive *versus* abortive trajectories.

In addition, the framework assumes that changes in fluorescence intensity faithfully reflect underlying molecular processes. Variability in fluorophore behavior, labeling efficiency, and photobleaching can introduce distortions in intensity traces, thereby affecting the accuracy of inferred growth rates. Photobleaching deserves particular attention, as it introduces an apparent negative component into intensity traces and can therefore bias inferred growth rates toward disassembly or reduced assembly rates. These effects are particularly relevant when comparing datasets acquired under different imaging conditions, where differences in laser power, exposure time, detector gain, fluorophore choice, and sample preparation can shift intensity scaling. While many of these issues also apply to single-particle tracking, careful matching of acquisition settings, background subtraction, normalization, and photobleaching correction are essential for cross-condition comparisons.

Spatial mapping further requires sufficient sampling to generate reliable statistics. In regions with low clathrin coat density or reduced signal-to-noise ratio, growth-rate distributions may be poorly constrained, leading to increased uncertainty in SD-based maps (Chenouard et al., 2014). This limitation is especially pertinent *in vivo*, where signal quality and particle density can vary substantially across space and time.

Deep learning–based extensions introduce additional considerations. Model performance depends strongly on the quality and diversity of the training data and may not generalize well to unseen conditions. Moreover, these models infer dynamics without explicitly incorporating mechanistic constraints, which can limit interpretability and hinder direct biological inference.

Overall, these limitations reflect a fundamental trade-off between robustness, scalability, and mechanistic resolution. Growth rate–based approaches provide a powerful and flexible framework for quantifying CME dynamics, but are best used in combination with complementary methods that offer detailed, event-resolved or molecular-level insights.

## Conclusion and future directions

Growth rate–based analysis has emerged as a robust framework for quantifying CME dynamics in living cells. By extracting local intensity derivatives from short temporal fragments rather than reconstructing complete trajectories, it captures population-level kinetics in regimes where frame-to-frame particle linking fails—including densely populated cellular regions, low-SNR conditions, and intact tissues such as the *Drosophila* amnioserosa. This robustness comes at the cost of event-specific information: questions involving individual coat lifecycles, diffusion, or productive-versus-abortive classification still require single-particle tracking, and the two approaches are best used in combination. The ability to extract kinetic information from short temporal windows (typically 12 s) and to generate spatial maps of endocytic activity at ∼8-µm resolution enables scalable analysis across both single cells and tissues. Recent advances, including deep learning–based inference of CME dynamics from static images, further extend this framework by approximating growth-rate SD maps from static images, eliminating time-resolved imaging at the inference stage while remaining dependent on growth-rate measurements for training.

Several promising directions can further advance this methodology. Integration with live-cell super-resolution microscopy may improve the spatial precision of clathrin measurements and enhance the fidelity of growth-rate estimates (Willy et al., 2021b; Akatay et al., 2022; Thompson et al., 2026). More broadly, extending this framework to other self-assembling systems—such as actin networks, membrane-associated scaffolds, and phase-separated assemblies—could reveal whether similar statistical descriptors capture dynamics across diverse biological processes.

An important future direction lies in the development of unified frameworks that link molecular-scale kinetics to cellular and tissue-level mechanics (Diz-Muñoz et al., 2013; Sheetz, 2001). Because growth-rate distributions and their associated metrics are sensitive to membrane tension and mechanical heterogeneity, they offer a potential bridge between biochemical dynamics and physical state. Coupling these measurements with controlled mechanical perturbations and quantitative imaging may enable direct interrogation of how biophysical forces regulate protein kinetics in living systems.

In summary, growth rate–based analysis provides a powerful and generalizable approach for quantifying protein dynamics under physiologically relevant conditions. By enabling minimally invasive, high-resolution measurements across spatial scales, it establishes a foundation for integrating experimental and computational strategies to connect molecular mechanisms with cellular function.
